# Epithelial alarmins at the crossroads of type 2 immunity: Experimental insights from skin and airway models 

**DOI:** 10.5414/ALX02604E

**Published:** 2026-10-22

**Authors:** Kexin Liao, Jamal Maurice Khani Maghanaki, Leonie Friederike Schotters, Yulin Zou, Anna Reidenbach, Katharina Klara Hahn, Prasad Dasari, Timo Buhl

**Affiliations:** Department of Dermatology, Venereology and Allergology, University Medical Center Göttingen, Göttingen, Germany

**Keywords:** epithelial alarmins, TSLP, IL-33, IL-25, type 2 immunity, epithelial barrier, atopic dermatitis, asthma

## Abstract

Epithelial alarmins such as thymic stromal lymphopoietin (TSLP), interleukin-33 (IL-33) and IL-25 orchestrate the initiation of type 2 immune responses at barrier surfaces. Dysregulated release of these cytokines links environmental cues to chronic allergic inflammation in the skin and airways. Recent experimental work using human keratinocytes, organotypic 3D skin equivalents, airway epithelial models, and murine challenge systems shows that barrier disruption, allergens, microbial components, and environmental irritants or pollutants induce strong alarmin expression. These mediators condition dendritic cells (DCs) and group 2 innate lymphoid cells (ILC2s), generating the cytokine milieu that drives Th2 polarization and neuro-immune crosstalk. This mini review summarizes molecular principles, highlights recent mechanistic findings from experimental systems, and discusses translational implications for allergic diseases such as atopic dermatitis, asthma, and chronic rhinosinusitis with nasal polyps (CRSwNP). Understanding the epithelial–alarmin axis offers opportunities for targeted prevention and therapy across barrier organs.

## Molecular principles of epithelial alarmins 

Epithelial barrier surfaces provide the first line of defense against environmental challenges and are equipped with rapid sensing systems that translate stress signals into immune activation. Among these systems, the alarmin cytokines thymic stromal lymphopoietin (TSLP), interleukin 33 (IL-33), and IL-25 act as early sentinels that bridge epithelial perturbation to adaptive immune responses. Each alarmin has a distinct molecular signature and receptor system: TSLP signals through a heterodimeric receptor composed of the thymic stromal lymphopoietin receptor (TSLPR) and IL-7Rα. IL-33 signals via a heterodimeric receptor composed of the IL-1 family receptor ST2 (IL1RL1) and the IL-1 receptor accessory protein (IL-1RAcP). IL-25 acts via the heterodimeric receptor IL-17RA and IL-17RB. Collectively, these pathways converge on NF-κB and STAT5 activation, resulting in the transcription of type 2 cytokines and costimulatory molecules that prime Th2 immunity [1, 2]. 

Figure 1 illustrates how epithelial alarmins orchestrate type 2 inflammation at the barrier surfaces of the skin and airways. When conditioned by TSLP, DCs upregulate OX40L and promote naïve T-cell differentiation into IL-4-, IL-5-, and IL-13-producing Th2 cells. Parallel to this adaptive route, the innate compartment contributes substantially through group 2 innate lymphoid cells (ILC2s), which respond directly to all three alarmins. Recent work has demonstrated a remarkable diversity and functional plasticity of ILC2 subsets, including tissue-resident and memory-like populations that retain heightened responsiveness upon secondary challenge [3, 4]. Despite overlapping effects, these alarmins differ in their target-cell profiles. TSLP promotes DC-mediated Th2 polarization, while IL-33 activates several type 2 effector populations, including ILC2s and basophils. IL-25 receptor expression is more restricted, with particularly high expression on ILC2s. The relative contributions of these pathways depend on the tissue and inflammatory context [2]. 

The influence of alarmins extends beyond immune cells and encompasses direct effects on sensory and structural components of the tissue. Protease-activated receptor-2 (PAR-2) signaling in keratinocytes facilitates bidirectional communication between the epidermis and peripheral neurons, contributing to pruritus and inflammation in atopic dermatitis [5]. Furthermore, neuronal IL-13Rα2 and IL-31R signaling integrates with epithelial cytokine networks to modulate itch and neurogenic inflammation [6]. These interactions highlight the integrative role of epithelial cytokines as coordinators of immune and neuronal activity. 

Beyond the classical alarmin triad, recent discoveries have expanded the concept of epithelial alarm signals (Table 1). TL1A (TNFSF15) has been identified as a constitutively expressed epithelial cytokine that synergizes with IL-33 to amplify IL-9-rich ILC2 responses, illustrating how multiple stress-responsive pathways converge to reinforce type 2 inflammation [7]. 

Experimental approaches to studying these interactions have evolved considerably. Quantitative PCR, ELISA and phospho-signaling analyses remain essential tools for identifying alarmin induction and downstream pathways. In parallel, organotypic human 3D skin equivalents containing immunocompetent cell lines and even primary immune cells now allow mechanistic exploration of keratinocyte and immune cell activation under well-defined environmental exposures [8, 9, 10]. Together with airway epithelial organoids and murine barrier-stress models, these systems form a powerful experimental platform for understanding epithelial-immune communication [11, 12]. Beyond the skin, epithelial alarmins also modulate airway sensory neurons, where IL-4/IL-13 and IL-33/TSLP-driven type 2 cytokines enhance cough reflexes and bronchoconstriction. 

## Cutting-edge findings across skin and airway models 

Emerging evidence demonstrates that common environmental chemicals and aeroallergens can directly modulate epithelial alarmin expression. Experimental data from murine models of atopic dermatitis show that the antimicrobial agent triclosan exacerbates disease severity through the upregulation of keratinocyte-derived TSLP, thereby establishing a mechanistic link between xenobiotic exposure and allergic inflammation [13]. Microbial stimuli, including *Staphylococcus aureus* membrane components, can also induce TSLP release from keratinocytes [14]. Similarly, topical sodium lauryl sulfate (SLS), long considered a prototypic irritant, elicits immune responses that differ fundamentally from those observed in classical irritant or allergic contact dermatitis [15]. In an HDM-induced murine airway inflammation model, intranasal HDM elicits canonical type 2 responses, with epithelial NOD1 signaling implicated as a contributing sensor that amplifies these outcomes [12]. These findings underscore that epithelial stress responses represent discrete immunological programs rather than uniform injury reactions. 

Allergens possessing protease activity, such as Der p 1, can also initiate epithelial activation by degrading junctional proteins and stimulating ATP/P2X and TLR signaling pathways. This disruption leads to IL-33 and TSLP release, which in turn instruct DC and ILC2 activation, demonstrating how allergen-specific enzymatic functions serve as built-in adjuvants for type 2 sensitization [16]. 

The use of immunocompetent 3D skin equivalents has further refined our capacity to dissect epithelial–immune interactions. These human-derived models faithfully reproduce alarmin release and subsequent DC activation following exposure to chemical or microbial stimuli [8, 9]. Such systems permit controlled investigation of dose–response relationships and facilitate mechanistic comparisons between toxic, irritant and allergenic stimuli. 

An EpiAirway full-thickness (FT) model and a normal human bronchial epithelial cell (NHBE)-based air-liquid interface (ALI) system have been leveraged to dissect epithelial stress triggered by environmental pollutants. Exposure to diesel exhaust particles (DEPs) further augmented the basolateral release of epithelial-derived inflammatory and profibrotic mediators, mechanistically linking airborne particulates to mucociliary failure, epithelial barrier injury and pro-inflammatory remodeling in the human airway [11]. Downstream of epithelial alarmins, IL-13 and IL-4 drive goblet-cell hyperplasia and MUC5AC overexpression, resulting in mucus hypersecretion and airway obstruction in asthma. 

Epithelial–neuronal communication represents another rapidly evolving area of research. PAR-2-dependent neuro-epidermal signaling and sensory neuron activation are increasingly recognized as amplifiers of inflammation in atopic dermatitis and other pruritic conditions [5]. Complementary in-vivo imaging studies have revealed structural neuronal alterations in the inflammatory skin disease rosacea, providing further evidence that epithelial barrier dysfunction and neural activation are intimately linked [17, 18]. 

Recent advances in human in vivo analyses have introduced skin microdialysis as a minimally invasive method for measuring local cytokine flux. This technique has already helped to identify disease-specific cytokine signatures in chronic inflammatory dermatoses and could be applied to capture real-time alarmin kinetics during allergen exposure or therapy [19]. 

These mechanistic insights integrate naturally into the broader “epithelial barrier theory”, which proposes that chemical pollutants, detergents, and microbial products damage epithelial integrity, induce alarmin release and thereby initiate Th2-dominant inflammation [20, 21]. While the core alarmin–ILC2–Th2 axis is conserved between skin and airway, tissue-specific DC subsets, structural cells (keratinocytes vs. bronchial epithelium), and the dominance of pruritus-driven neuro-immune loops in skin versus mucus and bronchoconstriction in the airway result in partially distinct effector patterns. 

### Clinical translation and therapeutic targeting 

The concept of epithelial alarmins provides a unifying explanation for the frequent coexistence of atopic dermatitis, asthma and chronic rhinosinusitis with nasal polyps (CRSwNP). In these disorders, epithelial cells act as upstream organizers of type 2 inflammation. Scratching, irritants, or pollutants trigger IL-33, IL-25, and TSLP release, which subsequently drive sustained activation of ILC2s and Th2 cells [22]. 

Therapeutic targeting of these upstream mediators has now entered clinical practice. The anti-TSLP antibody tezepelumab has demonstrated broad efficacy across diverse asthma phenotypes and long-term safety, as shown in the phase 3 NAVIGATOR trial and the DESTINATION extension study [23, 24]. The same molecule has proven beneficial in CRSwNP, where tezepelumab reduced polyp burden, improved quality-of-life scores and decreased the need for systemic corticosteroids in the phase 3 WAYPOINT trial [25]. Real-world studies, including the PASSAGE cohort, confirmed its effectiveness in routine care and across phenotypic subgroups [26, 27]. 

In the phase 2a FRONTIER-4 study, the primary endpoint was not met in the intent-to-treat population; however, positive efficacy signals were observed in a subgroup of patients with COPD at high risk of exacerbations [28]. Complementary mechanistic work has elucidated how IL-33 acts as a druggable hub connecting epithelial activation to systemic type 2 responses [29]. 

Although IL-25-specific therapeutics are not yet commercially available, experimental research supports IL-25 as a key damage-associated cytokine with potent ILC2-activating capacity [30]. Inhibition of this pathway may represent the next frontier of upstream anti-type 2 therapy. 

Beyond pharmacological interventions, alarmin biology suggests novel biomarker strategies. Measuring epithelial cytokine transcripts or proteins – either in serum, sputum, bronchoalveolar lavage, tissue biopsies, or via microdialysis – could provide sensitive indicators of barrier stress and treatment response [19]. The integration of such biomarkers into clinical trials would allow a mechanistic evaluation of therapeutic effects directly at the epithelial interface. 

From a practical standpoint, these insights support several strategies. Patients with barrier impairment should avoid exposure to surfactants and antimicrobial additives known to trigger alarmin release, such as SLS and triclosan [13, 15]. For individuals with multi-organ allergic inflammation, upstream biologics targeting TSLP or IL-33 offer a rational treatment approach. Furthermore, translational collaboration between basic immunology and clinical dermatology and allergology can accelerate the development of preventive measures by linking in vitro model systems with patient-derived data. 

Together, these advances illustrate how epithelial alarmins unify a mechanistic understanding across allergic and chronic inflammatory type 2 diseases. They represent both molecular sentinels of tissue stress and actionable targets for therapeutic modulation. Continued experimental research using sophisticated human models and translational biomarker studies will be pivotal in defining their roles in the next generation of precision medicine in allergy and clinical immunology. 

## Authors’ contributions 

K.L. and T.B. conceived the review and drafted the manuscript. J.M.K.M., L.F.S., Y.Z., A.R., K.K.H., and P.D. contributed to the literature review and critically revised the manuscript. K.L. prepared Figure 1. All authors reviewed and approved the final manuscript. 

## Funding 

No specific funding was received for this work.

## Conflict of interest 

None.

**Figure 1:**
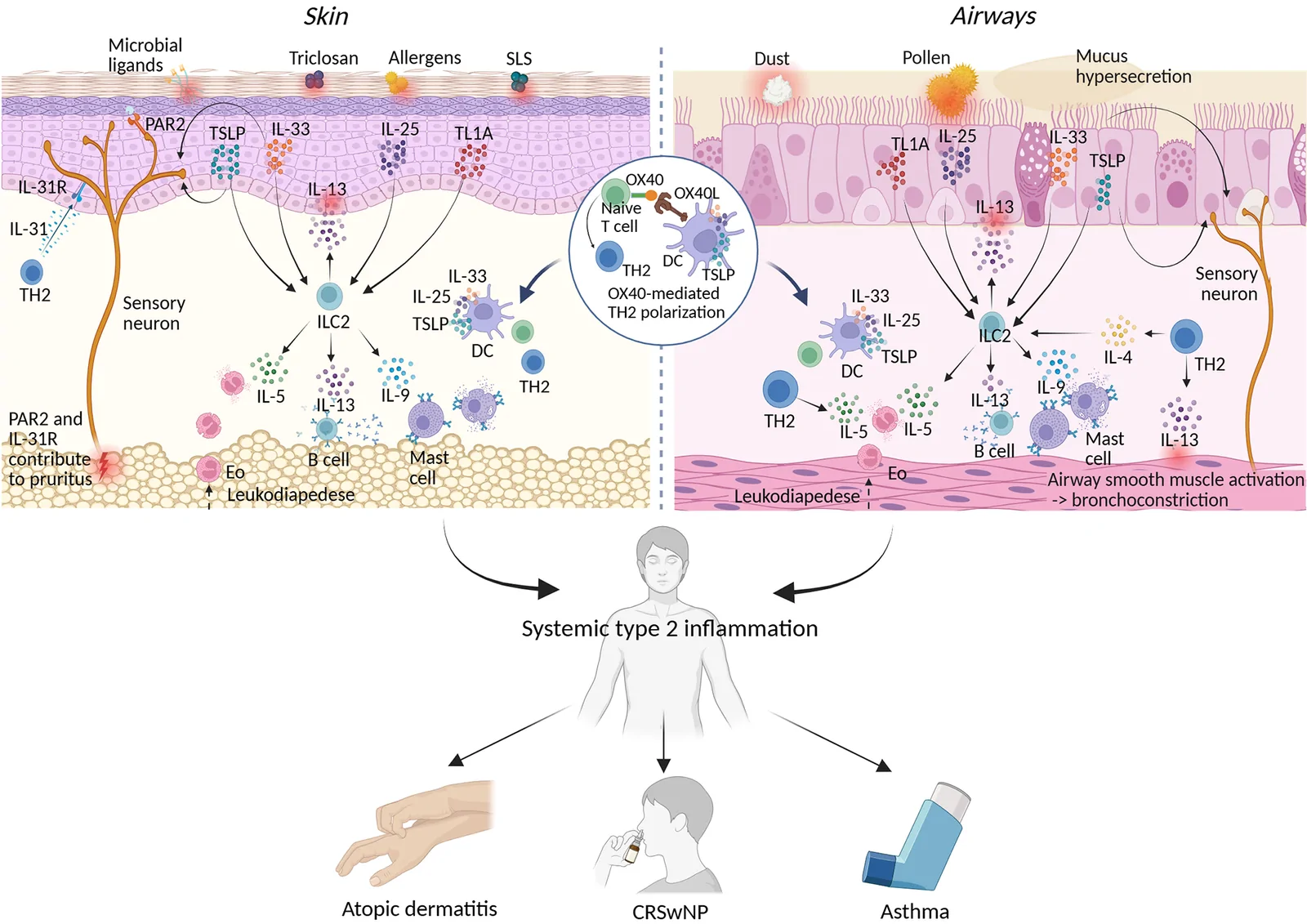
Epithelial alarmins orchestrate type 2 inflammation at barrier surfaces. This schematic integrates experimental findings from skin (left) and airways (right) to illustrate how epithelial stressors induce alarmin release, leading to DC conditioning, ILC2 activation, and type 2 inflammatory disease across barrier organs. Sensory neurons are shown in both tissues to illustrate shared alarmin-driven neuronal activation, although itch is restricted to skin, whereas cough and bronchomotor responses dominate in the lung. Figure was created with BioRender. ILC2 = group 2 innate lymphoid cell; TH2 = type 2 helper T cell; TSLP = thymic stromal lymphopoietin; TL1A (TNFSF15) = TNF-like ligand 1A (tumor necrosis factor superfamily member 15); DC = dendritic cell; Eo = eosinophil; PAR2 = protease-activated receptor 2; IL-31R = interleukin-31 receptor; ST2 (IL1RL1) = suppression of tumorigenicity 2 (interleukin-1 receptor-like 1); IL-1RAcP = interleukin-1 receptor accessory protein.

**Table 1.** Key features and translational aspects of epithelial alarmins.

**Alarmin**	**Receptor complex**	**Main target cells**	**Functional effects**	**Approved or advanced investigational therapies**
TSLP	TSLPR/IL-7Rα	DCs, ILC2s, neurons, murine basophils	Th2 polarization, itch, bronchomotor responses, murine basophil maturation and survival	Tezepelumab (approved); CSJ117 (phase 2)
IL-33	ST2/IL-1RAcP	ILC2s, Th2 cells, basophils, neurons	Type 2 amplification	Tozorakimab (phase 2); itepekimab (phase 2/3); etokimab (phase 2)
IL-25	IL-17RA/IL-17RB	ILC2s	Type 2 amplification	Experimental only
TL1A	DR3/TNFRSF25	ILC2s	IL-9 induction, synergy with IL-33	Tulisokibart (phase 3); PF-06480605 (phase 2)
